# Maternal–child consumption of ultra-processed foods and sugar-sweetened beverages in informal settlements in Mumbai, India

**DOI:** 10.1186/s41043-023-00486-z

**Published:** 2023-12-13

**Authors:** Aarti Kumar, Rachel J. Kulchar, Nehaa Khadka, Charlotte Smith, Piyasree Mukherjee, Erika Rizal, Karen Sokal-Gutierrez

**Affiliations:** 1 Berkeley School of Public Health, University of California, Berkeley, CA USA; 2 University of Pennsylvania School of Dental Medicine, Philadelphia, PA USA; 3 Los Angeles Fielding School of Public Health, University of California, Los Angeles, CA USA; 4 Swasti: The Health Catalyst, Bangalore, Karnataka India

**Keywords:** Ultra-processed foods, UPF/SSB, Sugary beverages, Nutrition

## Abstract

**Background:**

The global nutrition transition is associated with increased consumption of ultra-processed snack foods and sugar-sweetened beverages (UPF/SSB), contributing to the double burden of child obesity and undernutrition.

**Methods:**

This cross-sectional study describes the prevalence of maternal and child UPF/SSB consumption and the factors associated with frequent consumption in a convenience sample of 749 children ages 6 months through 6 years and their mothers participating in a community-based child oral health program in five informal settlement communities in Mumbai, India. Mothers were interviewed regarding maternal and child oral health and nutrition characteristics, including consumption of beverages and foods associated with tooth decay—milk, soda, tea with sugar, sweets, and chips/biscuits—using standardized questionnaires. Spearman correlations were used to assess for associations between various social factors and the frequency of maternal and child consumption of the five food categories. Chi-square tests were used to assess differences in child consumption patterns by age groups.

**Results:**

Though reported soda consumption was low among both mothers and children, nearly 60% of children consumed sweets and chips/biscuits daily, four to five times the rate of mothers. Factors associated with children’s frequent consumption of UPF/SSB included lower maternal education level, frequent maternal consumption of UPF/SSB, greater number of household members, greater amount of money given to the child, and closer proximity to a store.

**Conclusion:**

Our findings demonstrate social factors that may promote UPF/SSB consumption. The nutritional dangers of sugary drinks and non-nutritious snacks for mothers and young children should be addressed across maternal–child health, education, and social service programs. Early childhood nutrition interventions should involve the entire family and community and emphasize the need to limit children’s consumption of unhealthy foods and beverages from an early age.

**Supplementary Information:**

The online version contains supplementary material available at 10.1186/s41043-023-00486-z.

## Background

Over recent decades, trade liberalization, globalization, and widespread marketing of ultra-processed sugary beverages and snack foods have driven a global nutrition transition [1–6]. In India, snack food and beverage companies have sponsored sports and cultural competitions, and used social media, television, and celebrities to promote their products to young consumers [7]. From the 1970s to the early 2000s, India experienced increased availability and affordability of unhealthy foods, and this trend is expected to continue [1, 4]. As such, India, as well as most low- and middle-income countries, has experienced dramatic shifts in dietary practices from traditional breastfeeding and home-cooked meals to bottlefeeding and “Western diets” high in sugar, saturated fats, and highly processed ingredients [1, 5].

Increased consumption of non-nutritious snacks and beverages (i.e., “UPF/SSB”) [8, 9] has contributed to a “double burden of malnutrition,” with persistently high rates of undernutrition and obesity in India [10, 11]. The most recent national data on children under age five show that 38.4% experience stunting (41.2% rural, 31.0% urban), 35.8% underweight (38.3% rural, 29.1% urban) and 21.0% wasting (21.5% rural, 20.0% urban) [12]. Among school-age children, 9% are obese; and among urban South Indian adolescents aged 13–18, 18.5% of girls and 21.4% of boys are overweight or obese [13]. Moreover, during the COVID-19 pandemic, adolescent lifestyle habits were negatively impacted [14] and food insecurity and obesity prevalence were exacerbated [15], making the overconsumption of ultra-processed snack foods and sugar-sweetened beverages (UPF/SSB) an urgent concern [16]. A recent 2021 survey of Indian children from ages 9–14 reported that 93% ate packaged UPF/SSBs and 68% drank sugary beverages every week, including 53% consuming them one or more times a day [17]. Consuming UPF/SSB has been associated with increasing rates of tooth decay, child obesity, hypertension, and type II diabetes, especially among children in higher-income families [16, 18–20]. These consumption patterns have also been associated with undernutrition and vitamin deficiencies in children from low-income families by providing excess calories while lacking important micronutrients [21, 22]. Since many therapeutics to treat these diseases are prohibitively expensive [23, 24], it is critical to understand the social determinants that contribute to disease in order to prevent them.

With the persistence of child undernutrition and increase in obesity in India, there is a need to further examine the socioeconomic, educational, behavioral, and environmental characteristics that promote young children’s consumption of UPF/SSB and identify additional strategies to improve maternal–child nutrition interventions. The objectives of this study were to examine the frequency of consumption of UPF/SSB and to identify factors associated with frequent consumption in a sample of young children and their mothers from Mumbai, India.

## Methods

This is a cross-sectional study of maternal and child nutrition in a convenience sample of 749 children from age six months through six years and their mothers/caregivers participating in a community-based child oral health program, India Smiles, in five low-income informal settlement communities in and around Mumbai, India. Data were collected from December 2012 to December 2014 as part of a collaboration among University of California, Berkeley (UCB) and University of California, San Francisco (UCSF) with local non-governmental non-profit organizations (NGOs) in Mumbai, India: Foundation for Mother and Child Health (FMCH), Reality Gives, and Community Outreach Programme (CORP). All three organizations provide health, nutrition, education, social service, and vocational training programs to empower low-income community members, particularly mothers and children. Study approval was obtained from the Institutional Review Board (IRB) of UCB (#2012-11-4798) with IRB reliance from UCSF (#369-4) and from the board of directors of the partner NGOs. This research study was performed in compliance with the Helsinki Declaration.

The community health workers employed by the local NGOs invited families in the communities where they worked, with children in the designated age group, to participate in the program. The community health workers had trust with community members, substantial experience in maternal and child health and nutrition, and were well-trained to administer survey questions, obtain anthropometric measurements, and maintain confidentiality. Study personnel provided health workers with one additional day of training to administer the study survey and confirm procedures for measuring children’s length/height and weight, wearing light clothing and no shoes, with a stadiometer and digital scale (Seca, Chino, CA, USA), following WHO standards [25]. Each mother/caregiver was provided a written consent form and verbal explanation in her preferred language, and each child was provided simple verbal information for assent to participate. Trained community health workers and volunteers collected data through mother/caregiver interviews in the preferred language, with a survey of 50 questions modified from the World Health Organization (WHO) Oral Health Survey, on demographic characteristics and maternal and child oral health and nutrition, including consumption of selected beverages and foods associated with tooth decay—milk, soda, tea with sugar, sweets, and chips/biscuits (Additional file 1) [26]. This survey has been adapted and validated in studies in India [27], Vietnam [28], Nepal [29], Ecuador [30], and El Salvador [31]. Unique participant identification numbers with individual, family and site codes were assigned upon registration. Data were recorded on paper forms and manually inputted into Excel (Microsoft, Seattle, Washington), and R statistical programming software was used to merge datasets and select each child’s first visit. Data cleaning identified errors, inconsistencies, and outliers; corrected errors by utilizing data from the original hard-copy datasheets; and eliminated duplications and cases with critical missing data. The final dataset was exported to Stata SE17 (College Station, TX: StataCorp LLC) for analysis. Child nutritional status was determined using the WHO 2006 growth standards to calculate Z-scores for height for age (HAZ), body mass index for age (BAZ), and weight for age (WAZ). A low HAZ indicates stunting or chronic malnutrition, a low BAZ indicates wasting or acute malnutrition, and a low WAZ indicates underweight, which can be a combination of acute and chronic malnutrition [32]. Overweight was defined as BAZ >  + 2 but less than or equal to + 3 for children under age five, and BAZ >  + 1 but less than or equal to + 2 for children age five and older; obesity was defined by BAZ >  + 3 for children under age five, and BAZ >  + 2 for children age five and older [33–35].

For the descriptive analysis, we collapsed consumption patterns into binary variables, i.e., consumption “less often than daily” versus “one or more times per day.” For the association analyses, we used Spearman correlations, a nonparametric measure of the strength and direction of association between two variables measured on at least an ordinal scale, to determine the association between the ordinally reported maternal and child consumption of milk, soda, tea with sugar, sweets, and chips/biscuits; and the association between key sociodemographic factors with the ordinal frequency of maternal and child consumption of each respective food group. A descriptive analysis was completed for families’ demographic characteristics and mothers’ and children’s consumption patterns of milk (for mothers, animal milk; for children, breast milk, baby formula and animal milk), soda, tea with sugar, sweets, and chips/biscuits. Consumption of tea with sugar for both children and mothers as well as consumption of sweets for mothers was added to the standardized questionnaires in Year two. Hence, the sample sizes for these measures are smaller. Participants reported their frequency of consumption of each item on a scale from never consuming the food to having it two to three times per day. Chi-square tests were used to assess differences in child consumption by one-year age groups. For all tests, p-values of less than 0.05 were considered statistically significant.

## Results

This study involved 749 children from six months through six years old and 523 mothers/caregivers. If mothers had multiple children in this age group, they were all included in the study; specifically, 327 mothers had one child, while the other 196 had two to five children. The children’s mean age was nearly four years, and slightly more than half were male. Two-thirds of children were breastfed and not bottlefed, a quarter of children experienced mixed breast- and bottlefeeding, and 3.7% were bottlefed and not breastfed. For children who had reportedly concluded their period of breastfeeding and/or bottlefeeding (i.e., not still breastfeeding or bottlefeeding), the mean duration for breastfeeding and bottlefeeding was calculated: nearly 22 months for breastfeeding and 20 months for bottlefeeding. One in 10 children who were bottlefed drank sugary liquids in the baby bottle, including milk with sugar, tea with sugar, sugar water, soda, juice, and Horlicks (a popular, sweet, processed malted milk drink). In our study sample, there was a significant burden of chronic malnutrition with 40.1% experiencing stunting (low HAZ) and 40.7% underweight (low WAZ), with 15.6% experiencing wasting (low BAZ). Less prevalent were overweight (2.9%) and obesity (1.1%) (Table 1).

**Table 1:** Child, mother, and family characteristics

Characteristic	Mean (SD) or %			*N*^1^
*Child demographics*				
Age (years)	3.9 (1.6)			749
% Male	54.6			746
*Infant feeding practices*				
% Mixed breast- and bottlefeeding	25.2			729
% Breastfed only^2^	68.0			730
Duration of breastfeeding (months)	21.9 (10.5)			539
% Bottlefed only^2^	3.7			730
% Sugary beverages in baby bottle	10.0			211
Duration of bottlefeeding (months)	19.9 (13.0)			150
*Child nutrition status*^*3*^	*HAZ*	*BAZ*	*WAZ*	
% Undernutrition	40.1	15.6	40.7	729
% Overweight	–	1.0	–	729
% Obese	–	0.5	–	729
*Mother demographics*				
Age (years)	27.5 (5.1)			514
Education level (years)	6.4 (4.0)			516
Number of children	2.3 (1.1)			517
*Family characteristics*				
Number of household members	5.8 (2.3)			515
% giving children ≥ 10 rupees per day^4^	26.2			237
% living < 5 min’ walk to a store that sells UPF/SSB	81.0			515

^1^Though total sample consisted of 749 children and 523 mothers, reported N reflects sample available for each characteristic given missing data

^2^%Bottlefed only refers to children who were only bottlefed (not breastfed or mixed bottlefed/breastfed). % Breastfed indicates the percentage of children who were only breastfed (not bottlefed or mixed bottlefed/breastfed)

^3^Undernutrition by HAZ, BAZ, and WAZ is measured by a Z-score ≤  − 2. Overweight is measured as BAZ >  + 2 but less than or equal to + 3 for children under age five, and BAZ >  + 1 but less than or equal to + 2 for children aged five and older. Obesity is measured as BAZ >  + for children under age five, and BAZ >  + 2 for children aged five and older

^4^10 rupees was approximately $0.20 USD per 2012 conversion rate

The mothers had a mean age of 28 years, with a mean of six years of education. They had an average of two children, with a mean of approximately six people in the household. Over one in four families gave their children ≥ 10 rupees (approximately US $0.20) daily. More than four in five families lived less than a five-minute walking distance from a store that sold UPF/SSB (Table 1).

**Fig. 1:**
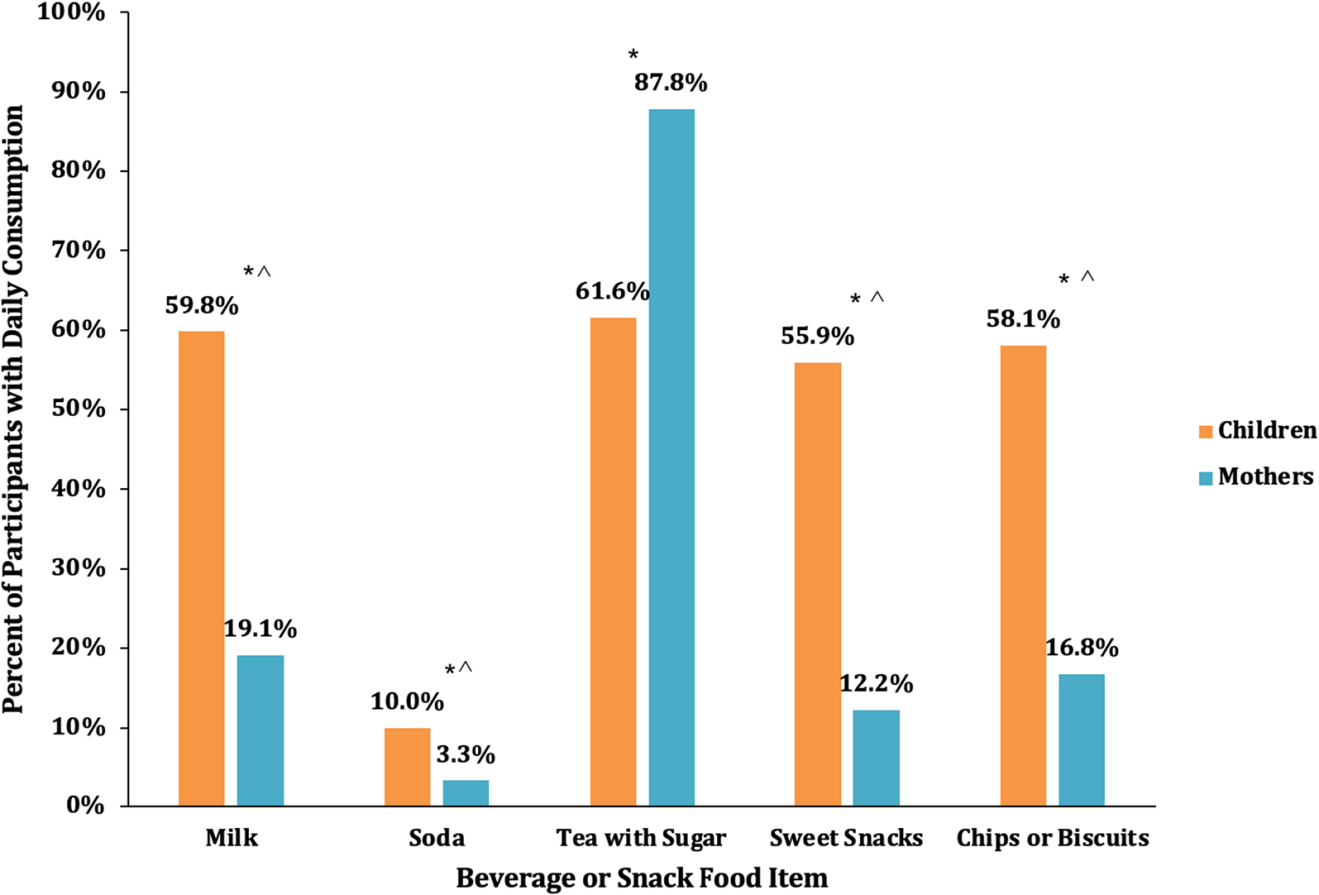
Maternal and Child Daily Beverage and Snack Food Consumption. *Spearman correlations for maternal and child consumption of all five food groups were statistically significant. Sample sizes varied due to missing data: child milk = 699; child soda = 721; child tea with sugar = 479; child sweets = 726; child chips or biscuits = 727; mother milk = 507; mother soda = 510; mother tea w/sugar = 336; mother sweets = 335; mother chips or biscuits = 513

Figure 1 displays the overall daily consumption of the specified beverages and snack foods by mothers compared to their children. Most mothers (87.8%) consumed tea with sugar daily, but only a small proportion of mothers consumed soda, sweets, or chips/biscuits daily. Children’s consumption patterns were distinct from their mothers. Overall, approximately 60% of children consumed each of the following daily: milk, tea with sugar, sweet snacks, chips/biscuits, while only 12–17% of mothers consumed these items; therefore, the percentage of children’s daily consumption of UPF/SSB was approximately four times as high as the percentage of mothers’ daily UPF/SSB consumption. The only item that was infrequently consumed was soda. Tea with sugar was the only item that mothers consumed more frequently than children. There were significant positive correlations between mothers’ and their children’s consumption pattern for both nutritious items (milk) and non-nutritious items (soda, sweets, chips/biscuits) (Fig. 1).

**Fig. 2:**
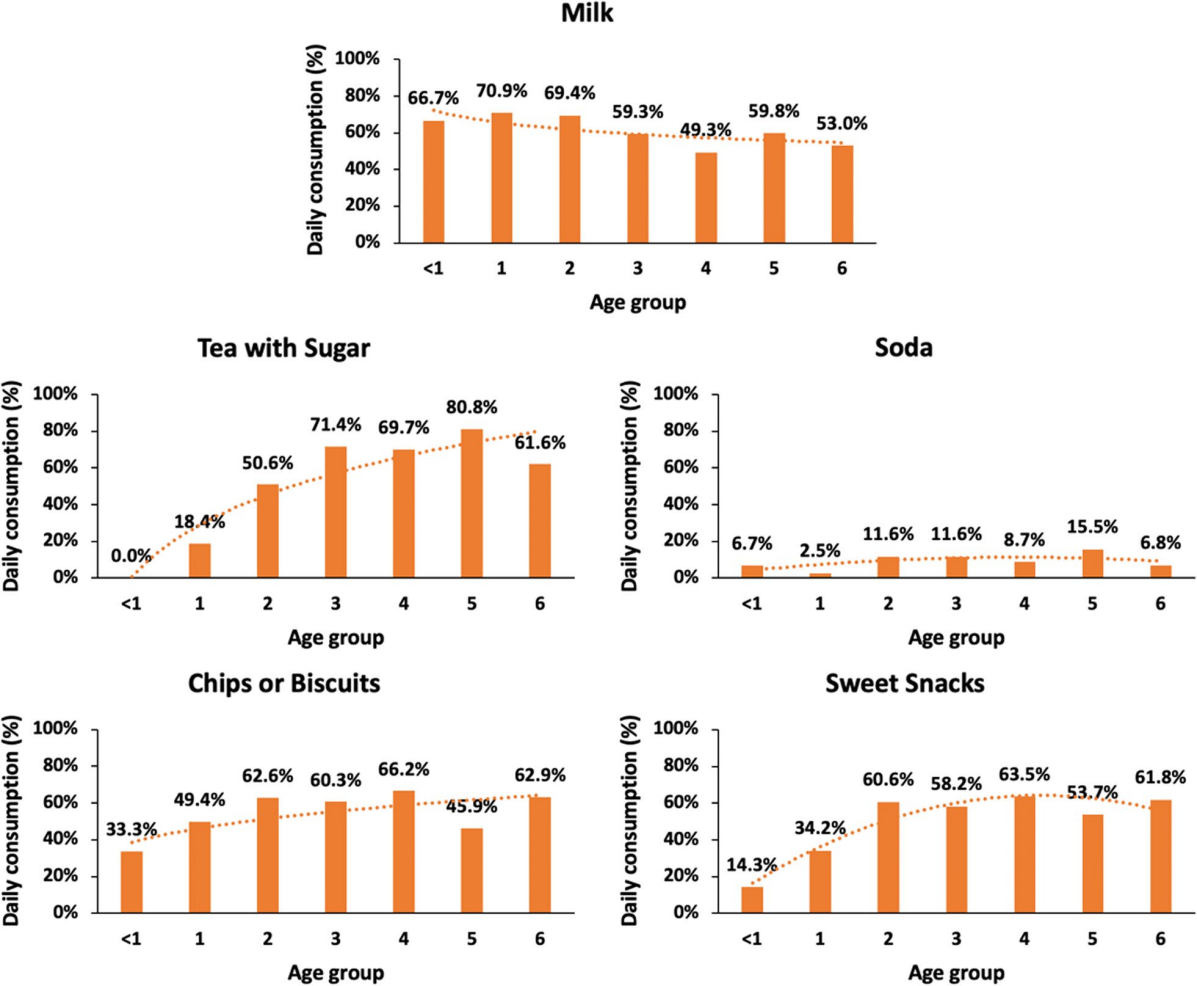
Children’s Daily Beverage and Snack Food Consumption by Age. *Chi-square tests showed significant differences in milk, tea with sugar, sweets, and chips or biscuits consumption by 1 year age groups. ^Sample sizes varied due to missing data: milk = 699; soda = 721; tea with/ sugar = 479; sweets = 726; chips or biscuits = 727

We also examined the frequency of children’s consumption of the five food categories across one-year age groups. With increasing child age from birth through age six, a general trend is seen for decreasing consumption of milk and increasing consumption of tea with sugar, sweets, and chips/biscuits. Importantly, under age one, a substantial proportion of infants had daily consumption of soda (6.7%), sweets (14.3%) and chips/biscuits (33.3%); by age two, a majority of toddlers had daily consumption of tea with sugar (50.6%), sweets (60.6%), and chips/biscuits (62.6%); and from ages three to six, children maintained a similar frequency of daily consumption of sweets and chips/biscuits, with increased frequency only in daily consumption of tea with sugar (Fig. 2).

When examining the sociodemographic factors associated with daily consumption of the specified beverages and snack foods, mothers' daily consumption of tea with sugar was positively associated with the mother’s education level and number of household members; no other associations were found (Table 2).

**Table 2:** Factors associated with mothers’ daily consumption of beverages and snack foods

Factor	Milk		Soda		Tea with sugar		Sweets		Chips/biscuits	
	r	*p* value	r	*p* value	r	*p* value	r	*p* value	r	*p* value
Mother’s age	0.002	0.972	− 0.026	0.637	0.106	0.058	− 0.062	0.268	− 0.021	0.713
Mother’s education level	− 0.025	0.652	0.039	0.489	**0.141**	**0.011**	0.109	0.051	0.042	0.453
Number of children	− 0.003	0.956	− 0.005	0.930	0.005	0.928	− 0.028	0.617	− 0.006	0.909
Number of people in the household	− 0.094	0.094	− 0.007	0.895	**0.128**	**0.021**	− 0.027	0.630	− 0.004	0.951
Proximity to UPF/SSB store (min)	− 0.086	0.125	0.030	0.598	0.048	0.390	0.028	0.624	− 0.081	0.147

Spearman correlation was statistically significant if bolded

**Fig. 3:**
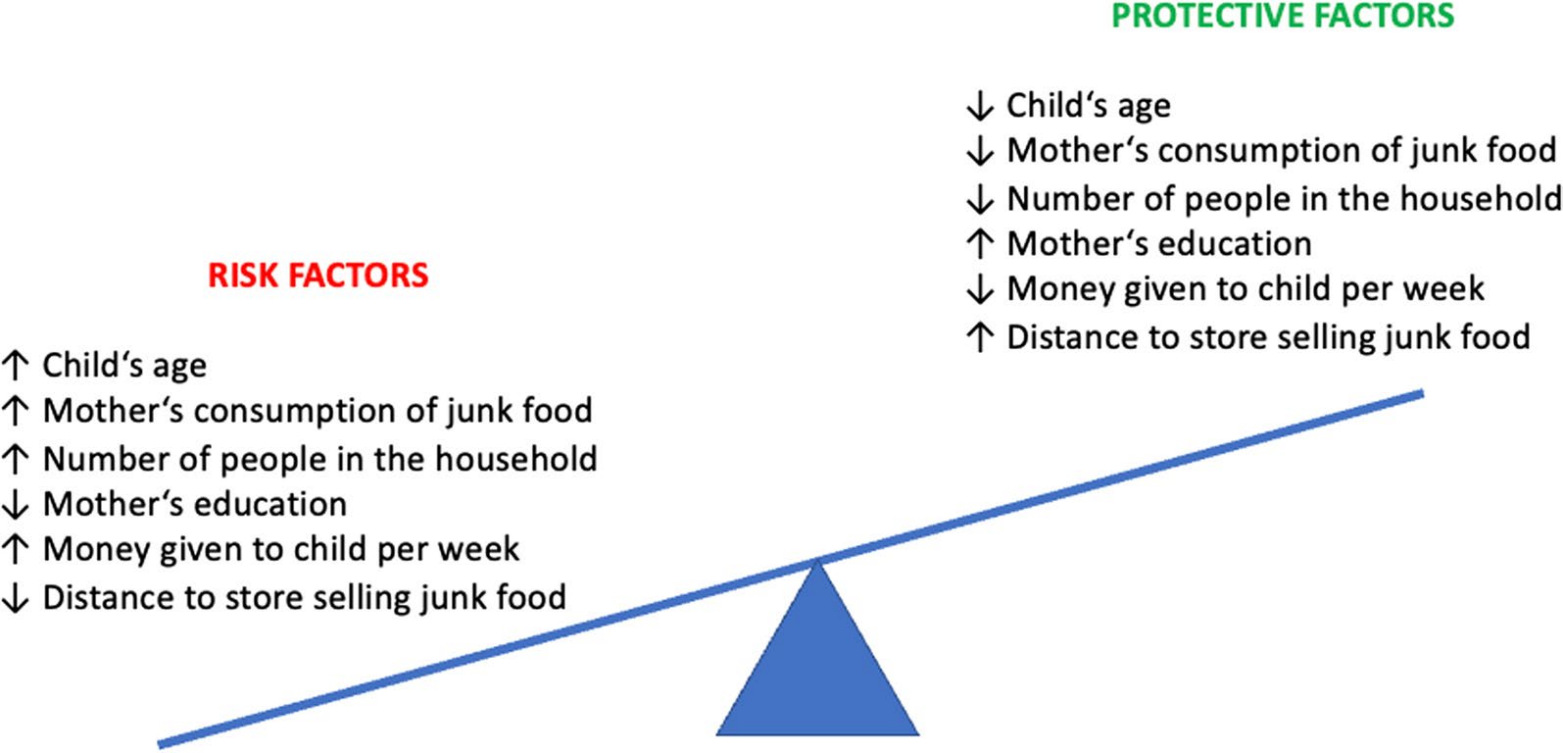
Protective Factors and Risk Factors of Increased UPF/SSB Consumption*. *Risk and protective factors were obtained from Table 3

In contrast, many sociodemographic factors were associated with children’s daily consumption of beverages and snack foods (Table 3, Fig. 3). Notably, higher maternal education was associated with less child daily consumption of tea with sugar as well as sweets and chips/biscuits. A higher total number of household members was associated with increased daily consumption of chips/biscuits. A greater amount of money given to the child was associated with greater daily consumption of soda and tea with sugar and chips/biscuits. Closer proximity to a store that sold UPF/SSB was associated with increased daily consumption of sweets. Though the child’s age was not correlated with consumption measures, chi-square tests demonstrated significant differences in milk, tea with sugar, sweets, and chips/biscuits consumption by one-year age groups (Fig. 2).

**Table 3:** Factors associated with children’s daily consumption of beverages and snack foods

Factor	Milk		Soda		Tea with sugar		Sweets		Chips/biscuits	
	r	*p* value	r	*p* value	r	*p* value	r	*p* value	r	*p* value
Child’s age	− 0.114	0.314	0.153	0.175	0.073	0.522	0.096	0.398	0.065	0.565
Duration breastfed	0.077	0.498	− 0.095	0.404	− 0.036	0.751	− 0.055	0.627	0.041	0.717
Duration bottlefed	− 0.096	0.399	− 0.192	0.088	0.093	0.413	0.129	0.256	− 0.046	0.683
Mother’s age	0.118	0.298	0.047	0.680	− 0.068	0.550	− 0.063	0.582	0.108	0.342
Mother’s education level	0.066	0.558	0.044	0.699	** − 0.256**	**0.022**	** − 0.276**	**0.013**	** − 0.300**	**0.007**
Number of children	0.039	0.734	0.131	0.225	0.111	0.328	0.096	0.399	0.215	0.055
Number of people in household	0.011	0.920	− 0.040	0.725	− 0.004	0.973	0.135	0.234	**0.228**	**0.010**
Money given to child	− 0.143	0.469	**0.457**	**0.015**	**0.395**	**0.038**	0.368	0.054	**0.488**	**0.008**
Proximity to UPF/SSB store	− 0.075	0.506	− 0.121	0.285	− 0.019	0.867	** − 0.227**	**0.043**	− 0.042	0.710

*Spearman correlation was statistically significant if bolded

## Discussion

This study found that in a community with a significant burden of chronic malnutrition, most young children consumed sugary beverages (particularly tea with sugar) and non-nutritious processed snack foods daily, demonstrating that ultra-processed foods and sugar-sweetened beverages have become a daily staple of young children’s diets in Mumbai informal settlements. Children’s daily consumption of UPF/SSB was approximately four times as high as the rate of mothers’ daily UPF/SSB consumption, indicating that the nutrition transition is occurring not only at a global level, but at the family level across one generation. Our findings are consistent with other studies that have found generational differences in UPF/SSB consumption within the same household [36, 37].

Snack food companies are known to target marketing to children and parents of young children [38], including assertions that chocolate, biscuits, and sugary drinks are healthy for children; make children happy, strong, and intelligent; and are a quick and inexpensive meal. This is particularly of concern as food consumption habits in childhood are likely to become lifelong dietary patterns [36]. Families with working parents, who have less time to prepare home-cooked meals, are particularly vulnerable to giving their children money to buy food, which often includes UPF/SSB [39, 40]. The finding that mothers consumed less UPF/SSB than their children may be a positive sign—those mothers valued their traditional, healthier diets. Moreover, studies show that parents help shape children’s eating preferences [41]. Nutrition education programs could build on mothers’ healthier eating habits and counter the advertising claims by emphasizing that sugary drinks and UPF/SSB interfere with children’s appetite and growth, and that children need traditional, natural, and nutritious food and beverages to optimize their growth, energy, and educational potential.

The many risk and protective factors associated with children’s frequent consumption of UPF/SSB demonstrate possible targets for intervention. A socio-ecological approach [42] can identify factors that could be addressed by interventions at maternal–child, family, community, and global levels. At the maternal–child level, the correlations between childhood UPF/SSB consumption with both mother’s UPF/SSB consumption and children’s age [43, 44] demonstrate that prenatal, postpartum *and* early childhood maternal–child health services should educate on the short- and long-term health hazards of eating non-nutritious snack foods and drinking sugary beverages. In the informal settlement communities included in our sample, we found that higher maternal education was associated with lower child UPF/SSB consumption, which is consistent with other studies [45, 46]. However, it appears there is a dual phenomenon in that UPF/SSB consumption and obesity are highly prevalent in affluent, well-educated families [47]. This suggests that while female empowerment and education initiatives may be helpful in low-income communities to promote socioeconomic mobility and perhaps the health status of the mother and her children, there are other factors in addition to parents’ education level that contribute to poor nutrition.

At the family level, the number of household members and money given to the child were significantly associated with children’s consumption of UPF/SSB and oral health status, underscoring the importance of extending interventions to the whole family, including grandmothers, who are traditionally key decision-makers for Indian household finances, parenting and child nutrition practices [27, 48]. At the community level, the strong association between proximity to stores and children’s UPF/SSB consumption supports findings that urbanization and global marketing have made unhealthy snack foods and beverages easily accessible in children’s environments. The Food Safety and Standard Authority of India (FSSAI) recently implemented the “Eat Right Movement “ which bans all UPF/SSBs and pre-packaged foods inside schools and within 50 m of the school campus [49]. This new movement can help limit children’s daily access to and consumption of UPF/SSB [50]. Additionally, expanding Indian regional taxes on UPF/SSB may prove successful as in Mexico [51], where increased prices of taxed beverages have led to lower sales and reduced consumption of unhealthy products [52]. Powell et al. assessed the outcomes of SSB taxes around the world and found that taxes to increase the price of sugary drinks by 20% would in turn decrease its consumption by 24% [53, 54]. Global nutrition advocates can collaborate to combat the economic infrastructure contributing to the global pandemics of malnutrition, obesity, and other non-communicable diseases. For instance, the Assessment & Research on Child Feeding (ARCH) Project’s work on the negative impacts of commercially produced foods have influenced the WHO’s guidance to end the promotion of UPF/SSBs for infants. Furthermore, advocacy by non-communicable disease and dental experts led to the development of WHO guidelines on limiting sugar intake for adults and children [55]. Additional nutrition policies and public education are needed to achieve Sustainable Development Goal 2, improving food security and nutrition.

This study is not without limitations, including convenience sampling, which may limit the generalizability of the findings; and cross-sectional design, which cannot establish causation. Our study focused on informal settlements in Mumbai. Therefore, the associations found may not be generalizable to other communities in India. In addition, dietary information was limited to self-reported measures of consumption of five food/beverage categories and did not include a complete maternal–child dietary record. Mothers may have underreported their children’s consumption of UPF/SSB to avoid judgment by the interviewer or because they were unaware of their children’s UPF/SSB consumption when unsupervised. In addition, while we explored a variety of social determinants, this list was by no means all-inclusive and there are surely other factors at play that we were unable to assess. We performed Spearman correlations to denote associations between two measures; however, this is an unadjusted relationship that does not account for potential confounders. The study’s strengths include a sizable study sample of children and mothers in low-income communities. In addition, we were able to evaluate both mothers’ and children’s consumption of UPF/SSB and sugary beverages as well as various factors associated with consumption patterns, which are often neglected in nutrition studies and interventions in developing countries.

## Conclusions

This study of children from six months through six years of age and their mothers in Mumbai, India found that over half of the children consumed UPF/SSB daily. This study found several maternal–child, family and community-level factors associated with children’s consumption of UPF/SSB and identified opportunities to incorporate nutrition education and policies to limit the consumption of non-nutritious snack foods and sugary beverages through maternal–child health services, schools, social services, and governmental regulations. Future studies are needed to demonstrate whether community and family interventions designed to target these factors can reduce young children’s consumption of UPF/SSB and improve their nutritional status and educational potential.

### Supplementary Information

**Additional file 1:** Study survey.

## Data Availability

The original datasets analyzed in this study are not publicly available in accordance with participant privacy, informed consent forms that did not include release of the data, and the study’s approved IRB protocols. However, the minimal dataset necessary to interpret, replicate, and build upon the findings of this study is available from the senior author [KSG] upon reasonable request.

## Abbreviations

ARCH: Assessment & Research on Child Feeding
CORP: Community Outreach Programme
FMCH: Foundation for Mother and Child Health
FSSAI: Food Safety and Standard Authority of India
IRB: Institutional Review Board
NGOs: Non-governmental non-profit organizations
UCB: University of California, Berkeley
UCSF: University of California, San Francisco
WHO: World Health Organization
