# Supplementary material for: Maternal–child consumption of ultra-processed foods and sugar-sweetened beverages in informal settlements in Mumbai, India
Source: J Health Popul Nutr. 2023 Dec 13;42:142. doi: 10.1186/s41043-023-00486-z (PMC10720234; doi:10.1186/s41043-023-00486-z)
Supplement: Supplementary file 1 — Additional file 1: Study survey. [file 41043_2023_486_MOESM1_ESM.pdf]

Mother Interview

(If the mother didn't do the interview, who did? Name, relation to child) :

ID:

Mother's Name:

Location:

Interviewer:

Date of Interview: day month year

1. Mother's age:

0 1 2 3 4 5 6 7 8 9

2. Mother's yrs of school completed

0 1 2 3 4 5 6 7 8 9

3. How many children does mother have?

0 1 2 3 4 5 6 7 8 9

4. How many are less than 7 years old?

0 1 2 3 4 5 6 7 8 9

Names of children less than 7 years old	Ages

5. How many people live in your house?

0 1 2 3 4 5 6 7 8 9

6. Does your house have electricity?

○ Yes ○ No

7. Does your house have potable water?

○ Yes ○ No

8. Do you cook with wood, gas or both?

○ Wood
○ Gas
○ Electric stove
○ Both

9. How long does it take you to walk to a store where you can buy snack food (e.g. chips, candy, soda)?

○ Less than 5 minutes
○ 6 to 20 minutes
○ 21 to 40 minutes
○ 41 to 60 minutes
○ More than 60 minutes

10. When do you go to the doctor?

○ Only when I'm sick
○ For check-ups
○ Both
○ Truthfully, I never go to the doctor.

11. When do you go to the dentist?

○ Only when I have problems
○ with my teeth/molars
○ For check-ups
○ Both
○ Truthfully, I never go to the dentist.

12. How often do you drink milk?

○ Never
○ Every 2-4 weeks
○ Every week
○ 2-3 times per week
○ Once a day
○ 2-3 times per day

12a. How often do you drink tea with sugar?

○ Never
○ Every 2-4 weeks
○ Every week
○ 2-3 times per week
○ Once a day
○ 2-3 times per day

13. How often do you drink soda?

- ◯ Never
- ◯ Every 2-4 weeks
- ◯ Every week
- ◯ 2-3 times per week
- ◯ Once a day
- ◯ 2-3 times per day

14. How often do you eat chips, biscuits, etc.?

- ◯ Never
- ◯ Every 2-4 weeks
- ◯ Every week
- ◯ 2-3 times per week
- ◯ Once a day
- ◯ 2-3 times per day

14a. How often do you eat sweets, candy, chocolate etc.?

- ◯ Never
- ◯ Every 2-4 weeks
- ◯ Every week
- ◯ 2-3 times per week
- ◯ Once a day
- ◯ 2-3 times per day

14b. How much money do you spend on snack foods per week?

- ◯ 5-15 Rupees/week/child (1-2 rs/day)
- ◯ 15-30 Rupees/week/child (2-4 rs/day)
- ◯ 30-50 Rupees/week/child (5-7 rs/day)
- ◯ 50-70 Rupees/week/child (8-10 rs/day)
- ◯ above 70 Rupees/week/child (>10 rs/day)

15. In your life, how many of your teeth or molars have fallen out or been extracted? (Not including baby teeth)

⓪ ① ② ③

⓪ ① ② ③ ④ ⑤ ⑥ ⑦ ⑧ ⑨

16. Right now in your home, do you have your own toothbrush?

◯ Yes ◯ No

17. How long ago was the last time you went to the dentist? (months)

(Never been = 00)

⓪ ① ② ③ ④ ⑤ ⑥ ⑦ ⑧ ⑨

⓪ ① ② ③ ④ ⑤ ⑥ ⑦ ⑧ ⑨

18. Why did you go to the dentist the last time?

- ◯ Pain in the tooth/molar
- ◯ Bleeding Gums
- ◯ Decayed tooth/molar
- ◯ Filling of tooth/molar
- ◯ Extraction of tooth/molar
- ◯ Check-up
- ◯ Because I went with my child

Other:

19. In the past 3 months, have you had any problem with your teeth, molars, gums or mouth? (mark all that apply)

- ◯ Pain or sensitivity
- ◯ Decayed or loose teeth/molars
- ◯ Bleeding Gums
- ◯ Inflammation of the mouth

Other:

(20-22, Don't prompt possible responses):

20. What do you think – what causes caries in baby teeth? (multiple answers ok)

- ◯ I don't know
- ◯ Sweets
- ◯ Gum
- ◯ Juice/Soda
- ◯ Not brushing your teeth
- ◯ Baby bottle

Other:

21. Do you think that caries cause problems for children?

◯ Yes ◯ No ◯ I don't know

22. How do they affect children?

- ◯ I don't know
- ◯ Pain
- ◯ They can't eat
- ◯ They can't sleep
- ◯ Decayed teeth/molars
- ◯ It harms their health

Other:

Child Information

ID:

24. Child's Name:

Mother's Name:

Interviewer:

25. Date of Birth:

day	month	year

25. Sex: Ⓜ Ⓕ

26. Did you receive prenatal care when you were pregnant with this child?

○ Yes ○ No

27. How many prenatal visits?

⓪①②③④⑤⑥⑦⑧⑨
⓪①②③④⑤⑥⑦⑧⑨

28. Are this child's vaccines up-to-date?

○ Yes ○ No ○ I don't know

29. Did you breastfeed this baby?

○ Yes ○ No

29b. If you breastfed, to what age (in months)?

⓪①②③④⑤⑥⑦⑧⑨
⓪①②③④⑤⑥⑦⑧⑨

29c. Still breastfeeding?

○ Yes ○ No

30. Did you give this child the baby bottle?

○ Yes ○ No

31a. If you gave the baby bottle, to what age?

⓪①②③④⑤⑥⑦⑧⑨
⓪①②③④⑤⑥⑦⑧⑨

31b. Still using baby bottle?

○ Sí ○ No

32. How often did he/she fall asleep with the baby bottle in his/her mouth?

○ Never
○ Occasionally
○ Frequently
○ Almost always

33. What did he/she drink in the baby bottle? (mark all that apply)

○ Water
○ Milk
○ Formula
○ Lemonade
○ Juice
○ Coffee
○ Soda
○ Sugar water
○ Tea (with sugar)

Other:

How often does this child consume the following things?:

34. Milk

○ Never
○ Every 2-3 weeks
○ Every week
○ 2-3 times per week
○ Once a day
○ 2-3 times per day

35. Soda

○ Never
○ Every 2-3 weeks
○ Every week
○ 2-3 times per week
○ Once a day
○ 2-3 times per day

35a. Tea with sugar

○ Never
○ Every 2-3 weeks
○ Every week
○ 2-3 times per week
○ Once a day
○ 2-3 times per day

36. Sweets/candy/chocolate

○ Never
○ Every 2-3 weeks
○ Every week
○ 2-3 times per week
○ Once a day
○ 2-3 times per day

37. Chips, biscuits, etc.

○ Never
○ Every 2-3 weeks
○ Every week
○ 2-3 times per week
○ Once a day
○ 2-3 times per day

(37-38, Don't prompt possible responses):

38. When your child cries during the day or the night, what do you do to calm him/her?

○ Comfort him/her
○ Hit him/her
○ Give him/her medicine
○ Give him/her sweets
○ Give him/her food
○ Give him/her the breast
○ Give him/her the baby bottle

Other:

38a. How much money do you give to your child per day?

○ 1-5 rupees
○ 6-10 rupees
○ 10-15 rupees
○ 15-20 rupees
○ more than 30 rupees

38b. What do you think your child spends the money on?

○ chips
○ candy
○ chocolate
○ sweets
○ soda
○ milk
○ toys

Other:

39. What do you do to take care of your child's teeth?

- ○ Brush them
- ○ Don't give candy
- ○ Nothing

Other:

40. Right now in your home, does your child have his/her own toothbrush?

○ Yes ○ No

41. Right now in your home, is there toothpaste?

○ Yes ○ No

42. Do you help your child brush his/her teeth?

- ○ Never
- ○ Occasionally
- ○ Frequently
- ○ Almost always

43. Has your child been to the dentist?

○ Yes ○ No

44. If he/she has been to the dentist, how many times?

⓪①②③④⑤⑥⑦⑧⑨

⓪①②③④⑤⑥⑦⑧⑨

45. If he/she has been to the dentist, why did he/she go?

- ○ For a check-up
- ○ Caries
- ○ Pain
- ○ Child has never been to the dentist

Other:

46. How often does your child complain of pain in the mouth/teeth/molars?

- ○ Never
- ○ Occasionally
- ○ Frequently
- ○ Almost always

47. How often does your child have problems eating because of mouth pain?

- ○ Never
- ○ Occasionally
- ○ Frequently
- ○ Almost always

48. How often does your child have problems sleeping because of mouth pain?

- ○ Never
- ○ Occasionally
- ○ Frequently
- ○ Almost always

49. In your opinion, how are your child's teeth?

- ○ Excellent
- ○ Okay
- ○ Bad

50. How is your child's health?

- ○ Excellent
- ○ Okay
- ○ Bad
